# An Effective and Simple Way to Establish Elastase-Induced Middle Carotid Artery Fusiform Aneurysms in Rabbits

**DOI:** 10.1155/2020/6707012

**Published:** 2020-08-26

**Authors:** Yanxia Lyu, Jie Luo, Yonghong Zhang, ChaoJia Wang, AnRong Li, Yi Zhou, EnFu Du, Hui Wang, JunTao Hu

**Affiliations:** ^1^Department of Physiology, Hubei University of Medicine, Shiyan, 442000 Hubei Province, China; ^2^Department of Neurosurgery, Taihe Hospital, Hubei University of Medicine, Shiyan, 442000 Hubei Province, China; ^3^Hubei Key Laboratory of Embryonic Stem Cell Research, Taihe Hospital, Hubei University of Medicine, China

## Abstract

**Objective:**

Elastase-induced aneurysms in rabbits have been proposed as a preclinical tool for device development, but there is still much deficiency in those aneurismal models. So we need to explore the efficient and convenient animal models for the investigation of intracranial aneurysms. Then, we compared and analyzed three methods of elastase-induced carotid artery aneurysms in rabbits and aimed to find a simple, effective, and reproducible method for creating elastase-induced aneurysms.

**Methods:**

42 standard feeding male adult Japanese white rabbits (3.05 ± 0.65 kg) were randomly divided into 3 groups and treated with elastase ablation to create common carotid artery (RCCA) aneurysm models: Group A (root-RCCA medication group, *n* = 12), Group B (mid-RCCA medication group, *n* = 18), and Group C (ligated RCCA+medication group, *n* = 12). For Group A, the origin of the RCCA was blocked by two temporary aneurysm clips, and the resulting 2 cm cavity was infused with elastase for 20 min, then the clip was removed and the RCCA was not ligated. For Group B, the middle part of RCCA was treated the same way as Group A and the RCCA was not ligated. For Group C, the middle part of RCCA was treated as Group B, but the distal RCCA was ligated. After the aneurysm models were created for 3 weeks, prior to sacrificing the animals, color Doppler ultrasound and angiography were performed for blood flow measurements inside the aneurysms. Histological analysis (such as SMA-*α*, CD31, CD34, CD68, collagen IV, and Ki67) and the other relevant indexes were compared between the ideal model's aneurysmal tissues and the human intracranial aneurysm's tissues to confirm whether we have successfully established elastase-induced aneurysm models.

**Results:**

Compared with human intracranial aneurysm specimens by the color Doppler ultrasound, angiography, and changes in the inner diameter of arteries, all three methods have successfully established the elastase-induced aneurysm models. Histology showed that biological responses were similar to both human cerebral aneurysms and previously published elastase-induced rabbit aneurysm models. Group A and Group B had the same morphology, but Group A had a higher mortality rate than Group B. Group B and Group C had different morphology. The aneurysm of Group C was more similar to human cerebral aneurysms but had a higher mortality rate than Group B. Group B was confirmed not only as an alternative method but also as a more safe and effective method for creating elastase-induced aneurysm models.

**Conclusion:**

Through analysis and comparison, the Group B is proven to be the simplest, reproducible, and most effective modeling method. The aneurysm model established by Group B can be used for basic research related to aneurysm mechanism. We have provided a new and effective method for basic research on aneurysm.

## 1. Introduction

Intracranial aneurysms (IAs) particularly influence the quality of a human's life, and their rupture is the most common cause of subarachnoid hemorrhage in adults [1]. IAs are characterized by an increase in external aortic diameter associated with fragmentation and degradation of elastic and collagen fibers, apoptosis of smooth muscle cells, and infiltration of inflammatory cells [2].

Animal models of IAs are useful for developing and evaluating ideally methods for treating aneurysms and help us to understand the mechanisms of IAs; thus, the models should closely resemble human aneurysms [3]. The rabbit RCCA aneurysm models are valuable for assessing devices for curing aneurysms, but most of the models require ligating the RCCA with silk sutures after creating the models, which is different with the natural aneurysms and may affect the blood supply to the brain [4]. Other common aneurysm models are the endoluminal application of elastase to rabbits, typically resulting in the aneurysm's morphology being similar to human aneurysms. Balloon occlusion and infusion catheters are used to intravascularly deliver the elastase solution to generate the aneurysm at the roots of the rabbit's RCCA. However, this method is expensive due to the need of endovascular interventional materials; in addition, one of the femoral arteries needs to be intubated and occluded, which may cause ischemic damage to the limbs [5–7]. The approach involves costly endovascular appliances and high doses of elastase (~100-200 U) [8–10]. However, the use of elastase is uncertain; there is another report wherein the doses of elastase do not need to be so high—10-30 U is enough [2].

Since there are still much deficiency and uncertainty in those previous aneurysmal models, we need to explore the efficient and convenient animal models for the investigation of intracranial aneurysms. Then, we compared and analyzed three methods of elastase-induced carotid artery aneurysms in rabbits and aimed to find a simple, effective, and reproductive method for creating an elastase-induced aneurysm.

## 2. Materials and Methods

### 2.1. Animals

The study was approved and conducted according to the guidelines set by the Experimental Animal Center of Hubei University of Medicine (China). All animal experiments were performed in the Experimental Animal Center according to protocols approved by the Animal Ethics Committee of Hubei University of Medicine. 42 standard feeding male adult Japanese white rabbits (3.05 ± 0.65 kg) were randomly divided into 3 groups and treated with elastase ablation to create common carotid artery (RCCA) aneurysm models: Group A (root-RCCA medication group, *n* = 12), Group B (mid-RCCA medication group, *n* = 18), and Group C (ligated RCCA+medication group, *n* = 12).

### 2.2. Rabbit Aneurysm Model Established [2–4, 11]

Animals were sedated with an intramuscular injection of 3% pentobarbital sodium (30 mg/kg). After complete anesthesia, rabbits were fixed in a supine position, neck hair was shaved, and iodine was applied for disinfection. Then, a longitudinal incision (~4 cm) was made along the anterior midline. The subcutaneous tissue, fascia, and sternothyroid were separated at the right side of the trachea to expose the RCCA but prevent dissection of the right vertebral artery (VA).

For Group A, the origin of the RCCA was blocked by two temporary aneurysm clips, and a 2 cm cavity between the two clips was created. Blood in the cavity was washed away with saline. Then, 20 units of pancreatic elastase (Shanghai Lin Ye Biotech Co., Ltd., China) were injected into the cavity and overflow was avoided. Twenty minutes later, the aneurysm clip was removed and the RCCA was not ligated. No blood leakage was observed, then the skin was sutured. Rabbits were returned to the Animal Experimental Center.

For Group B, the middle of RCCA (about 3 cm to the origin of the RCCA, but the RCCA bifurcation was not exposed) was temporarily occluded by two aneurysm clips. Then, 20 units of pancreatic elastase were injected, and the skin was sutured as was done with Group A.

For Group C, the middle RCCA was treated as was done with Group B, but the distal part of the RCCA was ligated.

To minimize postoperative infection in rabbits, animals received penicillin (North China Pharmaceutical Group Co., Ltd., China) (0.1 million units/kg, im, once a day) for 7 days postsurgery.

### 2.3. Visualization of Aneurysm and Measurements of Vessel Blood Flow

We selected 4 rabbits from each group and used color Doppler ultrasound to observe the aneurysms at 3 weeks postsurgery according to published methods [11]. Transcutaneous measurements of blood velocity of the LCCA and RCCA were obtained also.

### 2.4. DSA Examination of Induced Aneurysm

Digital Subtraction Angiography (DSA) was applied using a biplane DSA unit (GE, USA) 3 weeks postsurgery [11]. For DSA, animals were anesthetized and the femoral artery was exposed. Rabbits were prepared and DSA performed as described in published methods to observe the morphology of the aneurysm [12].

### 2.5. Histological Analysis of Elastase-Induced Aneurysms

After color Doppler ultrasound or DSA, external carotid arterial diameters were exposed and measured by a caliper. The models were then sacrificed by intracardiac injection of an overdose of sodium pentobarbital under general anesthesia (120 mg/kg). Arteries and aneurysms were removed after in vivo fixation with 10% phosphate-buffered formaldehyde solution. Each specimen was cut in consecutive slices of 2 mm from the proximal to the distal end and embedded in paraffin [2].

To validate the new model, we selected a giant human middle cerebral aneurysm and a renal artery to compare with the models' specimen. The sample of human saccular middle cerebral aneurysms was obtained from aneurysm clip operations at the Taihe Hospital (China), in one female, aged 53 years old. The sample of human renal artery was obtained from a nephroblastoma surgical operation at the Taihe Hospital (China), in one male, aged 61 years old.

Stain aneurysm specimens used conventional techniques and reagents with hematoxylin-eosin (H&E), *α*-smooth muscle actin (SMA-*α*), CD31, CD34, CD68, collagen IV, and Ki67 (Zhongshan Jinqiao Biotechnology Co., Ltd., Beijing, China). The primary antibodies used for rabbit aneurysm samples were mouse monoclonal antibodies, and the secondary antibodies are goat polyclonal anti-mouse (Zhongshan Jinqiao Biotechnology Co., Ltd., Beijing, China). The primary antibodies used in this section for human aneurysm specimens are rabbit monoclonal antibodies, and the secondary antibodies are goat polyclonal anti-rabbit (Absin Biotechnology Co., Ltd., Shanghai, China). The study protocol was reviewed and approved by the Institutional Review Board of Taihe Hospital, Hubei University of Medicine (China).

### 2.6. Statistical Analysis

Statistical analysis was performed using GraphPad Prism 6.07 for Windows (GraphPad Software, La Jolla, USA). Continuous variables were presented as means ± standard deviation and categorical variables as a number and percent. Comparisons of aneurysms among the 3 groups were performed using one-way ANOVA. Comparisons of aneurysms and LCCA diameters in the 3 groups were performed with Students' *t*-test (*P* < 0.05 was considered statistically significant).

## 3. Results

We have successfully created 50 aneurysms in 42 rabbits (not including the rabbits that died after anesthesia and before operation) as shown in Table 1, which include 32 fusiform aneurysms and 18 BBA (blood blister-like aneurysms). In Group B, there was no fusiform aneurysm found on one rabbit's RCCA, but there was one RCCA-BBA on it. The reasons may include the following: elastases have diverged which leads to insufficient damage to the artery wall and acupuncture to the artery wall causes local intimal damage. There are 3 BBA in Group A and 4 in Group B; those BBA could not be explicated by the puncture damage, so some BBA maybe the root of the broken branch artery. So the created aneurysm success rate only included the fusiform aneurysm but did not include the BBA. The 4 BBA right rabbits in Group A included the 2 BBA right and left rabbits. The 7 BBA right rabbits in Group B only included 2 BBA right and left rabbits. Operative death may be related to anesthesia and surgery damage. Postoperative death may be related to the damage of hypoxic-ischemic and surgical wound infection. DSA death may be related to anesthesia and blood loss during surgery.

All of the rabbits were dissected to verify the success of modeling. We could not locate the RCCA for two rabbits after modeling in Group A. One of the rabbits died due to unknown reasons. Aside from it being too late for us to know how the rabbit died, the blood clotted during the dissection process, making it difficult to find the RCCA. The other rabbit was executed uniformly with other models. The reason may be that the root of RCCA was seriously damaged and thrombosis had formed after modeling. Those reasons resulted in severe vascular ischemia leading to difficulty in finding and identifying RCCA.

BBA: blood blister-like aneurysms. The mortality rate does not include the rabbit death during ultrasound and angiography. Aneurysms included rabbits lost during anesthesia and surgery. The aneurysm success rate only included the fusiform aneurysm but did not include the BBA.

**Figure 1:**
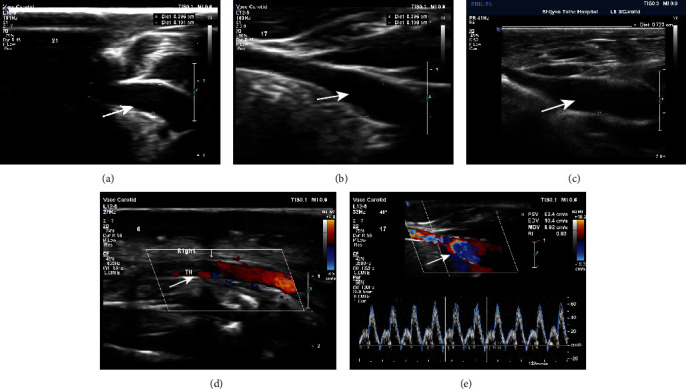
Color Doppler ultrasound demonstration of aneurysm creation. Samples were checked by color Doppler ultrasound. (a–c) B-mode: used to measure diameter. (a–c) refer to Group A, Group B, and Group C. Dist A = 0.396 cm, Dist B = 0.396 cm, and Dist C = 0.732 cm. (d) Doppler was used to measure arterial blood flow, TM pointed to by the arrow is the thrombus, and the red shows the blood movement. (e) Doppler was used to visualize flow inside aneurysm. PSV = 62.4 cm/s, EDV = 10.4 cm/s, and MDV = −8.92 cm/s.

**Figure 2:**
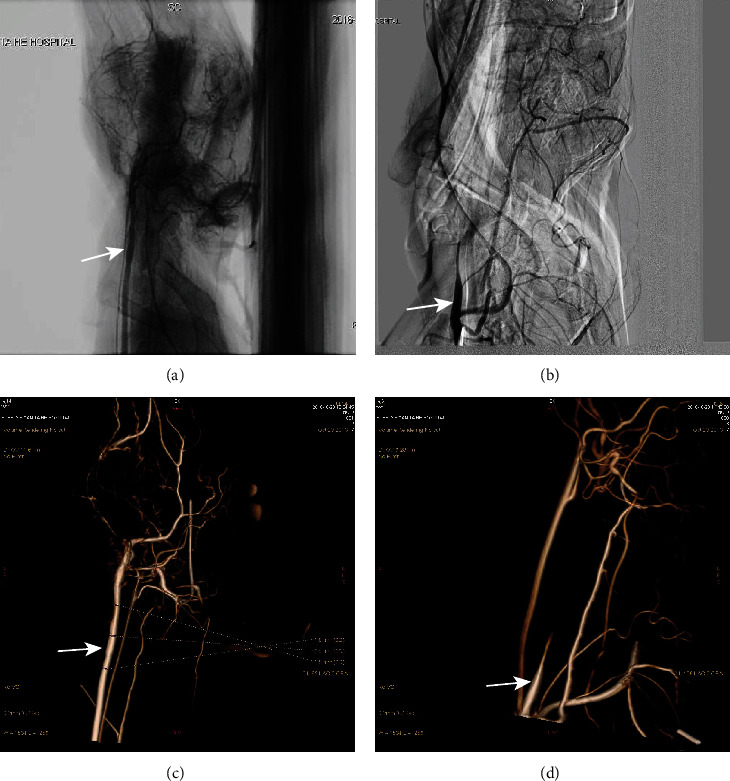
Angiographic demonstration of aneurysm creation. Samples were checked by DSA. (a) Fusiform aneurysm from Group B; angiographic of rabbit carotid artery 21 days after porcine pancreatic elastase incubation in the right carotid artery exhibited luminal enlargement (arrow) compared with that in the normal carotid artery. (b) Fusiform aneurysm from Group C with thrombus. (c) 3D results of Group B. (d) 3D results of Group C.

**Figure 3:**
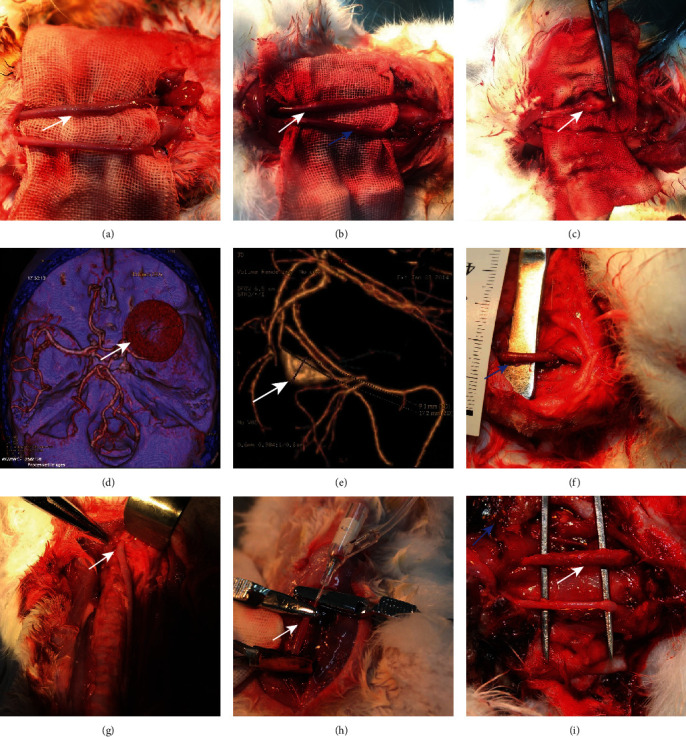
Photograph of aneurysm. (a) Group A fusiform aneurysm. (b) Group B fusiform aneurysm (white) and BBA (blue). (c) Group C aneurysm with thrombus. (d) Human saccular middle cerebral aneurysms. (e) Human fusiform middle cerebral aneurysm. (f) BBA. (g) Whole exposure of RCCA. (h) Temporary clip block to avoid elastase leakage. (i) Fusiform aneurysm (white) in rabbit and ulcer (blue).

**Figure 4:**
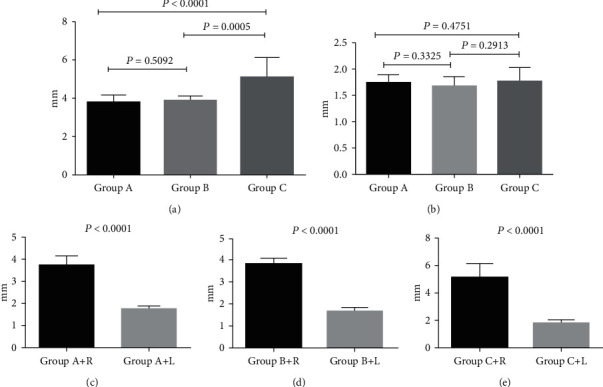
Morphometric results, bar graph representation of the morphometric results in elastase-treated carotid arteries (RCCA) compared with control untreated arteries (LCCA): (a) RCCA diameters compared among three groups (*P* < 0.0001). (b) LCCA diameters compared among three groups (*P* = 0.4751). (c–e) The RCCA diameters compared with the LCCA diameters separately in the three groups (*P* < 0.0001). Results are expressed as means ± SD.

Color Doppler ultrasound and angiographic of rabbit carotid artery 21 days after porcine pancreatic elastase incubation in the right carotid artery exhibited luminal enlargement compared with the normal carotid artery (Figure 1, Figure 2). There are some similar exterior characteristics between the Group C aneurysm and the human saccular middle cerebral aneurysms by physical observation (Figures 3(c) and 3(d)). Fusiform aneurysms in the rabbit have the similar appearance feature to human fusiform aneurysm (Figures 3(a), 3(b), 3(e), and 3(i)). The BBA could be found on the left and the right carotid artery (Figures 3(b) and 3(f)). The rabbit model with ulcer has a bigger fusiform aneurysm (Figure 3(i)). The morphometric results in elastase-treated carotid arteries (RCCA) were compared with control untreated arteries (LCCA) separately in the three groups (*P* < 0.0001), but the RCCA diameters were compared among the three groups (*P* < 0.0001), and the LCCA diameters were compared among the three groups (*P* = 0.4751) (Figure 4).

**Figure 5:**
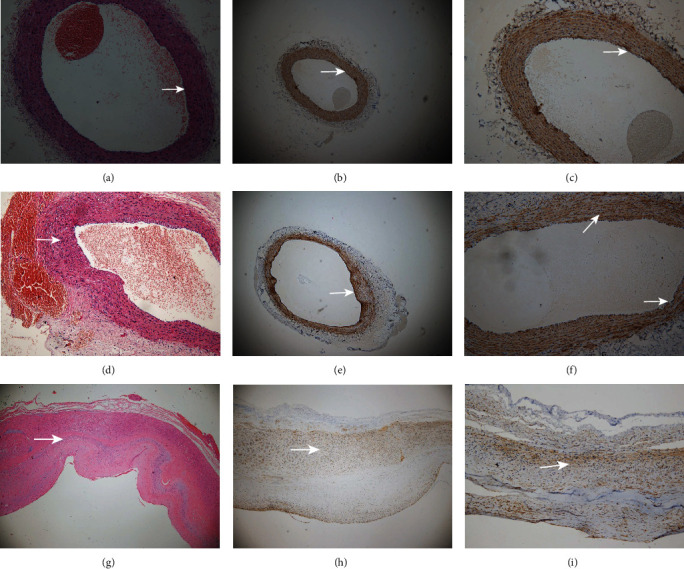
Pathological photograph of aneurysm and control arties. Example of control rabbit carotid artery (a–c): (a) H&E (10x), (b) SMA (4x), and (c) SMA (10x) showing normal control rabbit carotid artery. Example of rabbit carotid artery incubated with 20 U porcine pancreatic elastase (d–f): (d) H&E (10x), (e) SMA (4x), and (f) SMA (10x) showing maximal injury of elastin within elastase-induced aneurysm. Example of human aneurysm (g–i): (g) H&E (10x), (h) SMA (10x), and (i) SMA (10x) showing maximal injury of elastin within giant human middle cerebral aneurysms.

**Figure 6:**
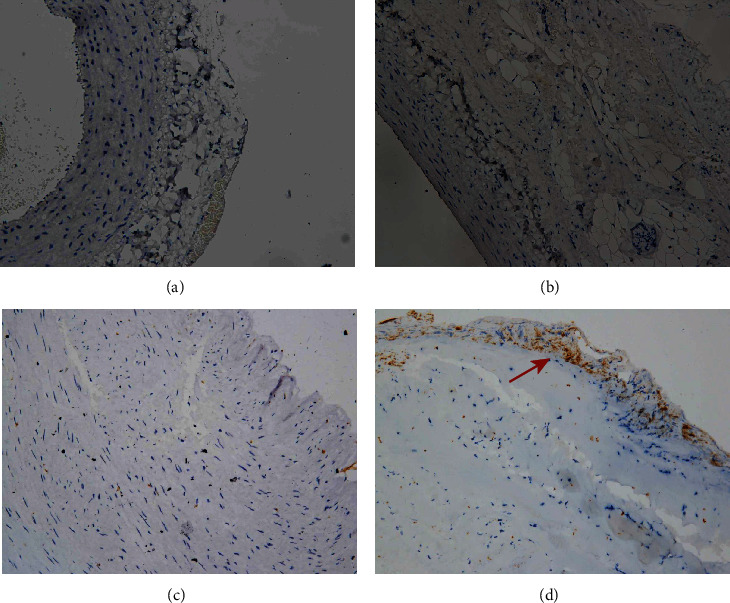
Immunohistochemistry. Arteries were stained with CD31. Example of control rabbit carotid artery (a), rabbit carotid artery incubated with elastase (b), human renal artery (c), and human aneurysm (d), ×20 (a–d). Positive cells are indicated by arrows (d).

**Figure 7:**
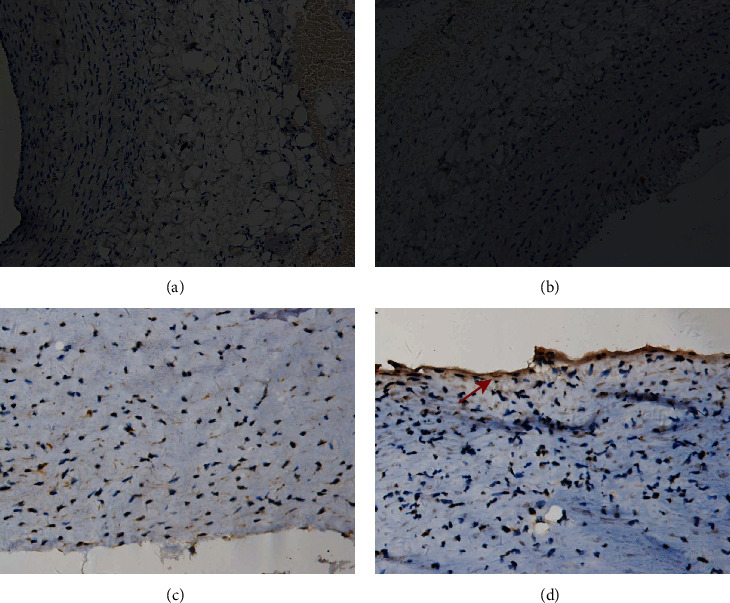
Immunohistochemistry. Arteries were stained with collagen IV. Example of control rabbit carotid artery (a), rabbit carotid artery incubated with elastase (b), human renal artery (c), and human aneurysm (d), ×20 (a–d). Positive cells are indicated by arrows (d).

**Figure 8:**
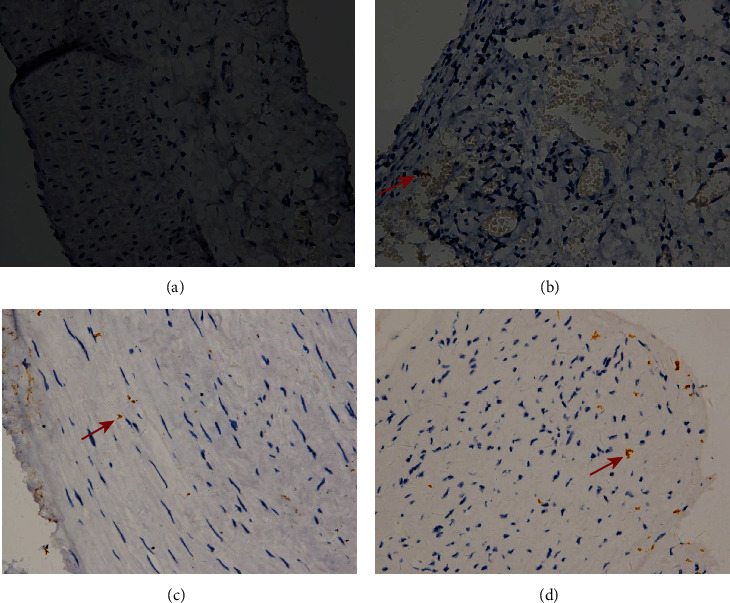
Immunohistochemistry. Arteries were stained with CD34. Example of control rabbit carotid artery (a), rabbit carotid artery incubated with elastase (b), human renal artery (c), and human aneurysm (d), ×20 (a–d). Positive cells are indicated by arrows (b–d).

**Figure 9:**
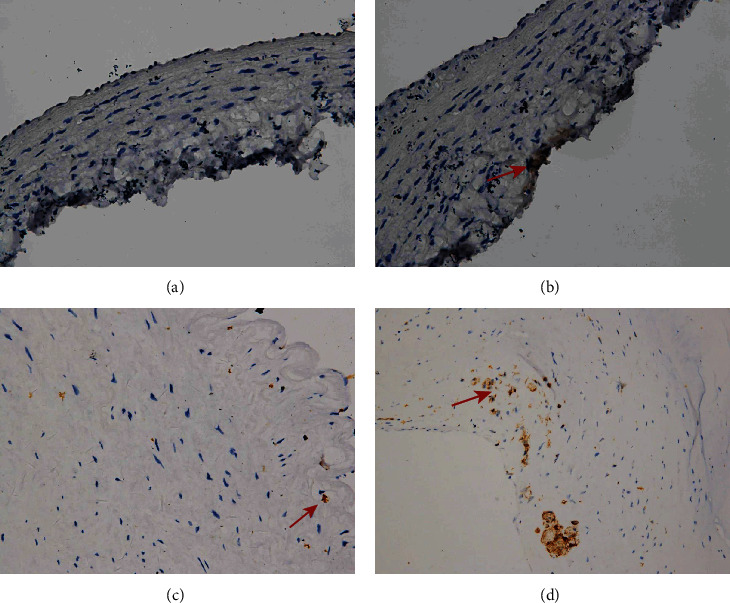
Immunohistochemistry. Arteries were stained with CD68. Example of control rabbit carotid artery (a), rabbit carotid artery incubated with elastase (b), human renal artery (c), and human aneurysm (d), ×20 (a–d). Positive cells are indicated by arrows (b–d).

**Figure 10:**
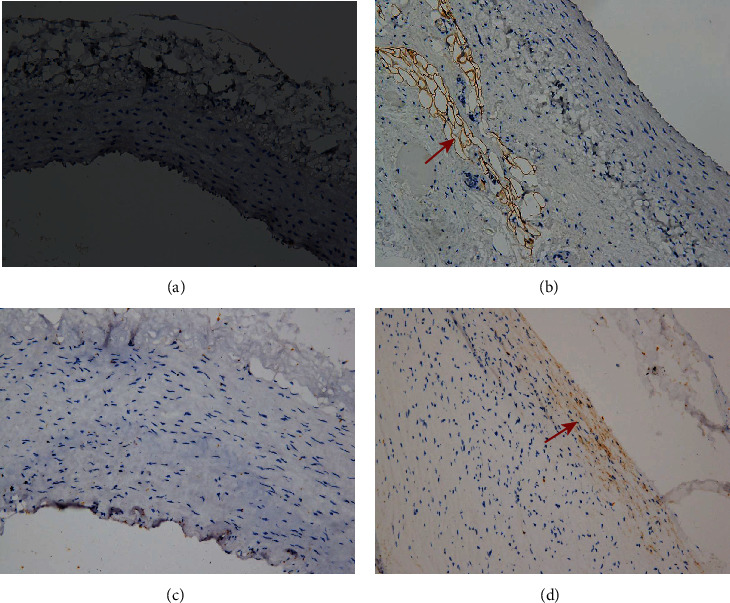
Immunohistochemistry. Arteries were stained with Ki67. Example of control rabbit carotid artery (a), rabbit carotid artery incubated with elastase (b), human renal artery (c), and human aneurysm (d), ×20 (a–d). Positive cells are indicated by arrows (b, d).

Smooth muscle cells were present in the media of the control rabbit carotid artery, elastase-induced fusiform aneurysms, and human aneurysm. There are some changes in the rabbit carotid artery after being incubated with porcine pancreatic elastase. All these changes in the elastase-induced fusiform aneurysms are similar to the structure of the human aneurysmal wall (Figure 5). CD31 and collagen IV were detected in human aneurysms, but not in control arteries and elastase-induced fusiform aneurysms (Figures 6 and 7). CD34, CD68, and Ki67 were detected in both human aneurysm and elastase-induced fusiform aneurysms, but not in control arteries (Figures 8, 9, and 10). CD34 is an important proinflammatory and proangiogenic cell in chronic inflammatory vascular disease [13]. The aneurysm tissues have some CD34-positive cell infiltration, while the control arteries have no significant CD34 positive cells, which prove that the characteristics of modeling aneurysms are consistent with those of normal aneurysms (Figure 8). There are a few of CD68-positive cells in the aneurysm tissue [14]. The CD68-positive cell rate in the elastase-induced aneurysms is significantly lower than that in normal aneurysms, proving that the inflammatory mediators affect the onset of aneurysms through monocytes/macrophages (Figure 9). Ki67 was positively expressed in both IAs and aneurysm models. Ki67 were detected in the media and adventitia of the elastase-induced fusiform aneurysms (Figure 10(b)), whereas Ki67 were predominantly detected in the neointima of the human aneurysm (Figure 10(d)).

## 4. Discussion

Elastase-induced aneurysms in rabbits have been proposed as a preclinical tool for device development [15, 16]. But the previous models have some limits, such as the need for expensive interventional materials, the femoral artery must ligated, and the high mortality rate. We aimed to develop a reproducible and easily accessible model of elastase-induced fusiform aneurysm in rabbit carotid arteries in order to mimic human IAs, making it possible to evaluate the medical treatment effect in the future.

We first concentrate our work on whether we could get the morphological features of aneurysm without using the interventional materials. We use three different methods to induce RCCA fusiform aneurysms in the rabbits by intravascular application of 20 U porcine pancreatic elastase. Histology, ultrasound, and DSA confirmed that our elastase-induced aneurysms were similar to those in previous reports [17, 18]. Our research showed that the fusiform aneurysms in the RCCA ligation group (Group C) were bigger than those in the other two groups. Size differences may be due to different hemodynamic conditions that led to increased interactions between blood flow in the parent vessel and the induced aneurysms [18]. Thrombus in Group C could influence the morphology of the aneurysm as well. In Group B, we did not find the fusiform aneurysm in one rabbit model but found a BBA on the RCCA. This may be due to the elastase not creating sufficient damage to the vascular wall in the rabbit; however, vascular wall punctures had damaged the local intima that lead to the BBA emergence.

We therefore investigated the different mortality rates of the three groups. Mortality of 5.6–33.3% has been reported in a study related to creating aneurysms by Japanese white rabbits. Technical modifications such custom intubation and capnostat monitoring of CO_2_ to adjust ventilation throughout surgery minimizes morbidity and mortality, and surgical experience is inversely correlated with mortality [19]. In our study, mortality was 33.3% in Group A, which could be attributed to operator experience, surgical auxiliary equipment, and range of operation. We reduced the surgery range by preventing exposure of the root of RCCA, then we have improved the survival rate in Group B and Group C. Mortality in Group C was 16.7% compared with 5.6% in Group B. The ligation of the RCCA that led to the reduced blood supply for cerebral may be responsible for the higher mortality in Group C.

Finally, we compared the model's fusiform aneurysms with human aneurysms by appearance feature and immunohistochemistry to verify the success of the modeling. Fusiform aneurysms in the rabbit have the similar appearance feature to the human fusiform aneurysm (Figure 3), although there are some different results between the aneurysm models and the human aneurysm after immunohistochemically dealing with the CD31 and the collagen IV. But in our study, H&E staining and SMA-*α* staining were used to compare the normal carotid artery, elastase-induced fusiform aneurysms, and human middle cerebral aneurysms. It was found that despite the smooth muscles' uneven thickness distribution, all these changes in the elastase-induced fusiform aneurysms are similar to the structure of the human aneurismal wall. The aneurysm tissues in Figures 8(b) and 8(d) have a small amount of CD34-positive cell infiltration, while the control rabbit carotid arteries have no significant CD34-positive cells, and the renal arteries have a few positive cell invasions, proving the molecular biological characteristics of the model to be consistent with the characteristics of human aneurysms. Figures 9(b) and 9(d) show a few CD68-positive cells in the elastase-induced fusiform aneurysms and human cerebral aneurysms, demonstrating that inflammatory mediators affect the onset of aneurysms through monocytes/macrophages. The Ki67 is related with rapid proliferation [20].  Figure 10 shows that the Ki67-positive expression is found in both IA and aneurysm models, but the expression concentration is different. Ki67 is mainly concentrated on the inner wall of the human aneurysm, while positive cells in the model groups are concentrated on the outer wall of the aneurysm, which may be different from the growth and development stages of the aneurysm. All of those demonstrate the success of our aneurysm modeling.

Our model allows for precise intravascular application of elastase and evaluation of flow diverter devices under various flow/morphological conditions. Flow diverting stents and other aneurysm embolization devices are evolving such as the magnetic resonance- (MR-) angiography techniques [21], low-profile visualized intraluminal support device [22], surpass streamline flow diverters [23], silk flow-diverter stents [24], and pipeline embolization devices [25, 26]. Our model allows quantifying the differences of thrombus organization within aneurysms with different morphologies and quantifying blood flow changes in the aneurysm. Our model uses less elastase and eliminates the need of endovascular devices as well as preserves the femoral artery for follow-up imaging/endovascular procedures. It therefore offers more success for inducing rabbit aneurysms.

There are some limits in our study such as our inability to apply ultrasound and DSA studies for all experiments to show changes in diameter and flow of the parent arteries. Secondly, we could not accurately measure aneurysm and vessel diameters. We had managed to characterize morphological changes of the RCCA, but we could not do the same for flow inside fusiform aneurysms. Finally, there were some differences in morphology and therapeutic measure between the elastase-induced fusiform aneurysms and normal IAs (such as the fusiform aneurysms in the cerebral territory) [27, 28], so this model will be better used to study the pathogenesis and the drug treatment of aneurysm, rather than the interventional treatment of fusiform aneurysms.

There are reports about generation of elastase-induced aneurysms in rabbits [18–30], but this represents the first attempt to explore a simple, effective, and reproducible method for creating elastase-induced aneurysms by comparing three different methods in rabbits. We have the lowest mortality (5.6% in Group B) and the similar success rate in creating aneurysms in the three methods, so Group B is the best recommended elastase-induced aneurysm method.

## Figures and Tables

**Table 1:** The results of aneurysm models.

Group	A(12)	B(18)	C(12)
Operative deaths	1	1	0
Postoperative death	3	0	2
DSA death	0	0	1
Mortality	33.3%	5.6%	16.7%
Fusiform aneurysm	9	13	10
Visible thrombus	0	0	3
BBA			
Right	4	7	0
Left	3	4	0
Right and left	2	3	0
Aneurysm	81.8%	82.3%	83.3%

## Data Availability

The data sets used and analyzed in the current study are available upon appropriate request from the authors.
